# Tissue macrophages: heterogeneity and functions

**DOI:** 10.1186/s12915-017-0392-4

**Published:** 2017-06-29

**Authors:** Siamon Gordon, Annette Plüddemann

**Affiliations:** 1 Graduate Institute of Biomedical Sciences, College of Medicine, Chang Gung University, Taoyuan City, 33302 Taiwan; 2 Sir William Dunn School of Pathology, University of Oxford, South Parks Road, Oxford, OX1 3RE UK; 3 Nuffield Department of Primary Care Health Sciences, University of Oxford, Woodstock Road, Oxford, OX2 6GG UK

## Abstract

Macrophages are present in all vertebrate tissues, from mid-gestation throughout life, constituting a widely dispersed organ system. They promote homeostasis by responding to internal and external changes within the body, not only as phagocytes in defence against microbes and in clearance of dead and senescent cells, but also through trophic, regulatory and repair functions. In this review, we describe macrophage phenotypic heterogeneity in different tissue environments, drawing particular attention to organ-specific functions.

**Electronic supplementary material:**

The online version of this article (doi:10.1186/s12915-017-0392-4) contains supplementary material, which is available to authorized users.

## Macrophages can be thought of as a dispersed homeostatic organ

Tissue macrophages constitute a distributed mononuclear phagocyte cellular system (MPS), contributing to the body’s responses to physiologic changes and to infectious challenge; thus, the MPS is comparable to the nervous and endocrine systems, in that it is adaptable, regulated and able to perform trophic [1] as well as defence functions, locally and systemically. Local macrophages induce tissue-specific metabolic responses such as hepatocyte biosynthesis of plasma proteins that provide an early response to infection in the acute phase reaction, and initiate features of systemic inflammation and infection such as loss of appetite and tissue catabolism [2]. The dual nature of macrophage functions, host protection versus tissue injury, is maintained in a fine balance; broadly, macrophage phagocytosis, clearance and secretion contribute to innate and adaptive defences against infection and underpin the process of inflammation, while the same processes, but with distinct secreted signals, restore tissue homeostasis and promote subsequent repair. Myeloid cells of the MPS interact with cells of the lymphoid system at many levels, recognition of non-self or modified self-antigens, initiating cellular and antibody immune responses, while executing effector functions which, if excessive or perpetuated, bring about tissue destruction. Monocyte migration and widespread tissue distribution provide portals for microbial dissemination, as well as host protection. During malignancy, tissue macrophages play an important role in promoting the survival, growth and spread of tumour cells [3].

Reflecting their ancient evolutionary origin, macrophage-like cells are found in many multicellular organisms, as motile, wandering cells performing a range of housekeeping, digestive and defence functions [4]. Even in their absence, in *Caenorhabditis elegans*, for example, other cells express comparable phagocytic functions. Elie Metchnikoff, immunology Nobel laureate of 1908 together with Paul Ehrlich, discovered macrophages in 1882 through experiments with simple marine invertebrates, where he recognized them as phagocytes able to respond to foreign particles and infection by a process analogous to inflammation in higher organisms [5]. This reputed “Eureka discovery” marked his transformation from comparative zoologist to experimental pathologist. His successors over the century since his death in 1916, appreciating that macrophages provided a widely distributed clearance system for particulates, coined the term reticulo-endothelial system (RES) for them—“reticular” because they are a network of cells, and “endothelial” because of particle uptake by sinus-lining intravascular cells [6]. This term was replaced by that of the mononuclear phagocyte system [7], to distinguish them from polymorphonuclear leukocytes and emphasise their specialised, although not unique, phagocytic prowess. In this review, we draw attention to their heterogeneity and broader trophic properties, conferred by the potential to express distinct sets of specialized surface and intracellular receptors that enable them to interact with other cells both locally and remotely, and support their viability, growth and specialised functions throughout the body, contributing to organogenesis and tissue repair.

The family of mononuclear phagocytes includes monocytes, macrophages, dendritic cells (DC) and osteoclasts, with common yet distinctive properties: distribution through multiple tissue compartments during development and adult life via blood and lymph; a common origin from haemopoietic stem cells and progenitors in specialised niches [8–10]; serving as sentinels of change and stress, being versatile and adapting to widely differing environments such as liver, gut, brain and bone. DC [11, 12] are specialised to process and present antigens to naïve lymphocytes at the initiation of adaptive immune responses [13], and osteoclasts are multinucleated giant cells which uniquely resorb living bone. The important functions of DC and osteoclasts are discussed in detail elsewhere [14, 15]: in this review we focus mainly on macrophages.

**Fig. 1.**
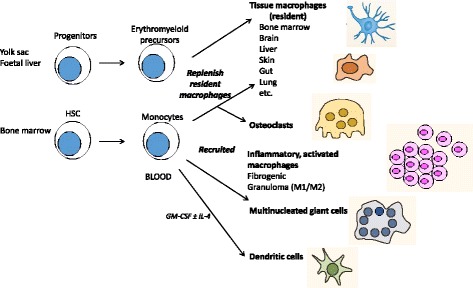
Origins and distribution of tissue macrophages. During development, erythromyeloid progenitors from yolk sac and foetal liver give rise to tissue-resident macrophages which persist during adult life as long-lived cells of widely varying mophology that turn over locally. Around the time of birth, bone marrow haemopoietic stem cells (*HSC*) become the source of blood monocytes, replenishing resident populations with high turnover, such as gut, and in response to increased demand. Therefore, different tissues contain varying mixtures of embryo and marrow-derived macrophages. In response to inflammation, immune and pathologic responses, monocytes infiltrate tissues and give rise to activated macrophages with complex phenotypes. Chronic immune cell aggregates can give rise to macrophage-rich granulomas, containing multinucleated giant cells as a result of monocyte/macrophage fusion. Monocytes contribute to osteoclast multinucleation and also generate functional dendritic cells upon culture in GM-CSF, with or without IL-4. Distinct monocyte populations give rise to DC [111], activated [111] and fibrogenic [18] macrophages

The origins, differentiation and heterogeneous fate of macrophages are schematically summarised in Fig. 1. During organogenesis, macrophages derived from embryonic yolk sac and foetal liver precursors are seeded throughout tissues, persisting in the adult as resident, self-maintaining populations, which turn over locally under steady state conditions and perform a variety of clearance and organ-specific trophic functions [16, 17]. After birth, bone marrow-derived blood monocytes replenish resident macrophage populations with high turnover, such as gut; larger numbers are recruited following injury, infection and sterile inflammation, and give rise to infiltrating, activated tissue macrophages. Organised macrophage-rich structures known as granulomas, for example, are formed in response to foreign bodies and chronic infections such as tuberculosis. Monocyte recruitment is also important in the host response to metabolic, atherogenic and neoplastic stimuli, contributing to wound repair and fibrosis [18, 19], angiogenesis [20] and tumour growth. Depending on the particular host location and requirements, tissue macrophages therefore consist of variably mixed populations of resident macrophages of embryonic origin and marrow-derived blood monocytes. As a result of their complex origin, distribution and biosynthetic responses to endogenous and exogenous stimuli, these cells express marked phenotypic heterogeneity.

Blood monocyte subpopulations also express phenotypic differences that reflect heterogeneity associated with their origin, maturation and activation [18, 21, 22]. They leave the circulation by squeezing through the blood vessel wall in a specialized process known as diapedesis, to give rise to heterogeneous tissue macrophages; or they can remain within blood vessels to help maintain the endothelium [23]. Distinct monocyte populations have been reported to contribute to fibrogenesis [18] and to myeloid-derived suppressor cells in malignancy [24]. Monocytes and macrophages express a wide range of surface, vacuolar and cytosolic molecules for recognition and uptake of host-derived and foreign particles by phagocytosis, and for clearance of soluble molecules by endocytosis [25]. They also produce a large range of secretory molecules, including neutral proteinases, chemokines, pro-and anti-inflammatory cytokines, and growth and differentiation factors, as well as low molecular weight peptides, and metabolites derived from oxygen, nitrogen, arachidonates and other lipids. Many of these properties and actions are induced in response to micro-organisms, which activate complex changes in gene expression. As well as responding directly to microorganisms, macrophages are activated by cytokines secreted by the lymphocytes of the adaptive immune system, which, with other environmental immunomodulators, can either direct macrophage differentiation into classic (M1) activation, with enhanced antimicrobial, inflammatory and antigen-presenting properties, or promote an alternative activation phenotype (M2) characterized by anti-inflammatory actions and a distinct set of antimicrobial actions (Additional file 1). These distinct phenotypes are induced by the actions of cytokines produced by two of the major classes of lymphocytes. The TH1 lymphocyte product interferon gamma induces the M1 phenotype, whereas the cytokines produced mainly by TH2 lymphocytes, interleukins 4 and 13, promote the M2 phenotype. It is widely recognised that the M1/M2 terminology is simplistic and that macrophage activation most likely reflects a spectrum of changes rather than a binary division [26]. Classically activated macrophages are characteristic of intracellular infections and bystander tissue injury, such as during tuberculosis; its failure during HIV-1 infection is associated with opportunistic infections, giving rise to AIDS. Alternative activation is associated with allergy, parasitic infection, repair and fibrosis.

**Fig. 2.**
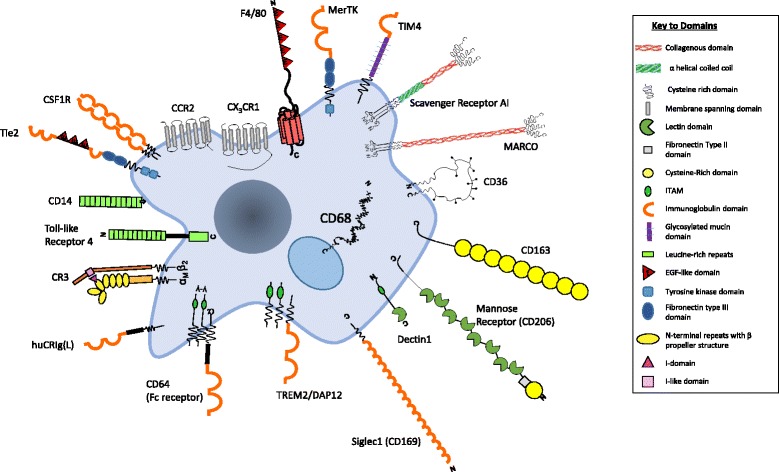
Selected plasma membrane receptors that mediate macrophage recognition of microbial and host ligands. Macrophages are able to express a large repertoire of membrane receptors implicated in the recognition and uptake of foreign and modified self ligands, some of which are illustrated here. These receptors incorporate a range of structural domains, illustrated schematically; they serve as useful marker antigens for immunocytochemistry and FACS analysis (e.g. F4/80, CD68, CSF1 receptor, Mer-TK, CD64). They function as opsonic (antibody and or complement coated particles to enhance uptake via Fc and complement receptors) or non-opsonic, carbohydrate-binding lectins and scavenger receptors. The phagocytic receptors mediate clearance of microbes (e.g. MARCO), apoptotic cells (for example CD36, SR-A, TIM4) and circulating ligands; for example, CCR2 and CX3CR1 are receptors for the monocyte/macrophage chemokines MCP-1 and fractalkine, respectively, for growth promoting and regulatory cytokines, for example, CSF-1 and angiopoietins, (Tie-2), and CD163 for clearance of injurious haptoglobin–haemoglobin complexes. Toll-like receptor-4 and CD14 react with bacterial membrane components such as lipopolysaccharide (LPS) to induce pro-inflammatory signalling; Dectin-1 recognises fungi through beta glucan in their wall, activating a range of innate immunological responses. Siglec-1 (CD169), a receptor for sialic acid terminal glycoconjugates, mediates adhesion of host cells and microbes, whereas CD206, a receptor for clearance of Mannosyl terminal glycoproteins, is a prototypical marker of M2 activation. The scavenger receptor SR-A internalises polyanionic ligands such as modified lipoproteins, as well as selected microbes, whereas CD36 mediates adhesion and M2-induced macrophage fusion and giant cell formation. TREM-2 mutations have been implicated in neurodegeneration and osteoclast dysfunction (see [25] and text for further details)

Building on this brief overview, we consider aspects of the adaptation of selected macrophages to particular tissue micro-environments and their role in specific organ and tissue functions. There has been a flurry of recent excellent reviews dealing mainly with the origin of resident tissue macrophage populations and the contributions of recruited monocytes during inflammation, infection and malignancy [16, 27–34]. However, we still have little insight into the mechanisms that determine their tissue differentiation and their contributions to tissue-specific functions. Figure 2 illustrates some of the diverse array of surface receptors whereby macrophages recognize microorganisms and host molecules, and that reflect the diverse functions discussed in this review.

## Macrophage heterogeneity can be identified in situ by differentiation antigens, fate mapping and gene expression patterns

**Fig. 3.**
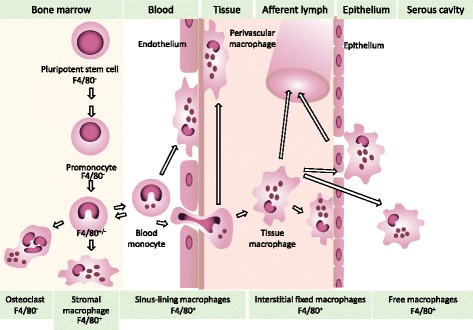
Schematic illustration of F4/80 antigen expression by tissue-resident macrophages in the mouse. Monocytes and macrophages express F4/80 antigen after differentiation and proliferation of F4/80 negative precursors in the embryo (not shown) and bone marrow. Mature F4/80+ macrophages associate with endothelia and epithelia as they migrate through tissues. Monocytes (+/-) replenish F4/80+ tissue-resident macrophages, for example in gut, liver, skin and brain, and contribute to formation of F4/80-negative osteoclasts. Macrophages lining lung alveoli and in T-cell-rich lymphoid tissues express F4/80 weakly. See Gordon et al. [112] for further details

Traditionally, the identification of macrophages in tissues depended on morphology, histologic staining and intravital labelling with phagocytic particles. The development of monoclonal antibodies to label membrane antigens selectively expressed on murine macrophages made it possible to detect their precise location and obtain evidence of heterogeneous antigen expression in different organs [35]. The F4/80 antigen [36] was particularly useful to map their presence in different body compartments of the mouse [37]. Figure 3 illustrates the expression of F4/80 antigen in bone marrow, blood and tissues. These studies revealed the close association of F4/80+ macrophages with neighbouring cells, facilitated by the exquisite plasma membrane-restricted expression of this antigen marker and its stability to fixation. In particular, F4/80+ macrophages associate with endothelia and epithelia, in addition to widespread interstitial distribution within organs and connective tissues. Morphology and expression of F4/80 and other antigens (Additional file 2) demonstrated marked microheterogeneity of tissue macrophages within, as well as among, different organs shown, for example, by microglia and macrophages in the central nervous system, as illustrated in [38]. In situ analysis underlined the importance of microanatomical niches in promoting phenotypic diversity and functional specialisation in precise tissue microenvironments.

Fate mapping and extensive microarray, enhancer and proteomic analysis established precursor-product relationships and gene expression phenotypes in tissue macrophages ex vivo. This has made it possible to identify common groups of proteins that are expressed together and are characteristic of all or specific specialized macrophages isolated from different sources [39]. These studies are consistent with known differences among tissue macrophages in different organs and have made it possible to discover new functions.

Tissue macrophage populations in the adult mouse are of mixed embryonic and bone marrow monocyte origin in the steady state and after inflammatory and infectious stimulation. Table 1 summarises the subpopulations of tissue-resident macrophages present in selected individual organs and their functions; Table 2 illustrates the characteristics of tissue macrophages derived from recruited monocytes in selected pathologies. We have chosen representative tissues in this review, to illustrate the complex heterogeneity and functions of both resident and activated macrophages, rather than an exhaustive review of all tissues. It is important to note that morphology and in situ immunocytochemistry reveal striking microheterogeneity within individual organs, only partially revealed by ex vivo analysis of extracted cell suspensions.

**Table 1:** Microheterogeneity of selected tissue-resident macrophages: phenotype and functions

Source	Macrophage subpopulation	Functions	Comments
Foetal liver	Stromal mϕ	Definitive erythropoiesis	Adhesion receptor distinct from phagocytosis
		Enucleation of erythrocytes	
	Foetal monocytes		
Bone marrow	Monocytes	Monocytosis	Release controlled by CD169
	Stromal mϕ	Haemopoietic islands, phagocytosis of erythroid nuclei	Sialoadhesin (CD169) and integrin mediate selective adhesion
		Haemopoiesis: erythroid (Fe recycling), myeloid (PMN, monocytes, eosinophils); plasma cells	
	Osteoclasts	Bone remodelling	Mutinucleated giant cells CSF1, RANK ligand, vacuolar ATPase, polarised adhesion; F4/80^−^, CD68^+^
		Osteoblast interactions	
Spleen	Red pulp	Clearance of senescent erythrocytes, PMN	F4/80^+^, CD206^+^
		Haem catabolism, Fe recycling	Induction Spi-C transcription factor
	Marginal zone, metallophils	Sinusoidal—clearance of polysaccharides, antigens, microbes; stimulate migration	CD169^+,^ CSF-1 dependent
	Outer marginal zone	Phagocytic	MARCO^+^, SIGNR-1^+^, type I interferon induction
	White pulp	Clearance of apoptotic T and B lymphocytes	F4/80 negative, CD68^++^
		Migrating metallophils transfer antigen to DC, which migrate to white pulp to activate T and B cells	
Lymph nodes	Subcapsular macrophages	Analogous to marginal zone	
		Afferent lymph delivers DC and antigens and viruses to lymph node	Subcapsular sinus macrophages capture antigens for delivery to DC, for activation of B and T lymphocytes
	Medulla	Activation of T and B cells	Filter for mϕ which do not enter efferent lymph
Gut	Lamina propria mϕ	Interaction microbiome, epithelium, innate (ILC2/3) and acquired lymphocytes	Active migration beneath epithelium of villi, sample the lumen
		Modulation of inflammation and immune activation	TGFbeta (F4/80, oral tolerance)
	Submucosal mϕ	Interactions with smooth muscle cells, myenteric and autonomic nervous system	Peristalsis
Peritoneal cavity	Large and small resident mϕ; elicited and activated	Interactions B1 lymphocytes. Inflammation stimulates migration to draining lymph nodes and abdominal organs, such as liver, upon injury	Prototypic in vivo inflammation model Reservoir of mature GATA-6^+^ macrophages for repair
Liver	Kupffer cells	Sinusoidal, clearance, phagocytosis and receptor-mediated endocytosis, interactions with hepatocytes, acute phase synthesis through contact and cytokines. Metabolism: iron, lipids, micronutrients. Immune desensitisation	F4/80^+^, CR3 dim, Kupffer cell-specific CRIG and other lectins. Clearance through CD206, SR-A, also by sinusoidal endothelium
Lung	Alveolar mϕ	Particle clearance	F4/80 dim, CR3 dim, CD206^+^ MARCO^+^, SR-A^+^
		Surfactant metabolism (type 2 alveolar cells)	GM-CSF, PPAR gamma
		Immunosuppression by activated alveolar macrophages	
	Bronchial mϕ and DCs	Antigen capture and presentation	
Heart	Resident cardiac macrophages in AV node	Regulate cardiomyocyte electrical activity through macrophage Connexin43- mediated adhesion	Interruption causes heart block
	Resident macrophages in myocardium of foetal origin, bone marrow-derived monocytes and macrophages sourced from other tissues	Response to myocardial ischaemic infarct, repair and tissue remodelling	Heterogeneous origin, including extramedullary haemopoiesis and proliferation, mediated by sympathetic nervous system
Large arteries	Tissue resident macrophages supplemented by monocytes	Monocyte adhesion to endothelium, interactions with lipids, smooth muscle cells, foam cell formation	Response to shear force. Atherogenesis and its complications (Table 2)
Brain	Microglia	Interaction with neurons, live and apoptotic	Resident microglia of yolk sac origin; F4/80^+^, CR3^+^ Can be supplemented by bone marrow-derived macrophages
		Sculpting of synapses via CR3, development and repair; interactions with axons and astrocytes	Microglial activation and complement production contribute to astrocyte activation and neurotoxicity
	Perivascular mϕ	Clearance lectins and SRs	CD206^+^, SR-A^+^
	Choroid plexus mϕ	Cerebrospinal fluid secretion	
	Meningeal mϕ	Clearance, lymphatic drainage?	Network

See text for references

Given the complexity of tissue-resident populations and admixture of recruited monocytes in the steady state and stress, it is important to study properties in situ. These and other organs, not included in this review, also contain interstitial macrophages (not included above)

**Table 2:** Phenotype of monocyte-derived tissue macrophages in selected pathologies

Disease process	Monocytes/macrophage characteristics	Comment	Reference
Inflammation/infection	Ly6C+, FcgammaRIII+. Distinct precursors give rise to iNOS+, CD209a-, MHC- microbicidal macrophages and CD209+ MHCII+ monocyte-derived DC	GM-CSF induces differentiation of distinct GM and MDC progenitors	[111]
Th1-mediated granuloma formation (e.g. Tuberculosis)	Classic M1 activation (IFN gamma), epithelioid transformation (adhesion molecules), foam cells (lipid storage), Langhans giant cells (DC-STAMP fusion) Cell necrosis, caseation, cavitation (metalloproteinases) and fibrosis	iNOS+ PMN-recruited and metabolic switch	[116, 117]
TH2 cell-mediated granuloma formation (e.g. Schistosome eggs)	Alternative M2 activation (IL-4/13), multinucleated giant cells (CD36-mediated) and fibrosis (TGF beta)	TGM2+, arginase, upregulation of CR3 function. Metalloproteinases, metabolic switch, eosinophils and mast cells	[118, 119]
Bleomycin-induced fibrosis	Atypical fibrogenic monocytes (SatM) arise from Ceacam1+ SR-A+ Ly6C- F4/80- Mac1+ precursors	Arise from Ly6C- Fc ϵ R1 granulocytic/macrophage progenitors, licensed by C/EBPbeta	[18]
Atherosclerosis	Monocyte and platelets adhere to altered endothelium, foamy macrophages (cholesterol and apolipoproteins) and migrating smooth muscle cells Macrophages promote plaque rupture, coagulation and embolism	CSF-1 upregulates SR-A and metalloproteinases	[120]
Cancer:			
Tumour-associated macrophages (TAMs)	Tumour attracts monocytes, macrophages contribute to tumour growth and angiogenesis	CSF-1. F4/80 promotes immune tolerance	[3]
Myeloid-derived suppressor cells (MDSC)	Abnormal differentiation of monocytes and granulocytes. Immunosuppression	Essential metabolite consumption. Reactive oxygen and nitrogen, display inhibitory surface molecules to alter T-cell trafficking and viability	[24]
Metastasis-associated macrophages (MAM)	Macrophages promote intra- and extravasation and survival of the tumour cells. Monocytes are Ly6C+ F4/80+ CD11b + CCR2+ Flt-1hi Tie2hi VEGF+	Flt-1 signalling, CSF-1 pathway, FAK(p), MAPK(p)	[121]

## Stromal macrophages promote and support erythropoiesis

In mouse foetal liver, stromal macrophages take part in definitive erythropoiesis, from day 10, reaching a peak at days 13–14, before declining at birth as the bone marrow takes over. Recent studies by Gomez-Perdiguero and colleagues have shown that foetal liver macrophages are generated from yolk sac erythro-myeloid (EM) progenitors, independent of myb, a transcription factor required for adult haemopoietic stem cells (HSC). The colony stimulating factor-1 (CSF-1) is a macrophage-specific growth and differentiation glycoprotein, and its receptor, also known as oncogene c-fms, is widely expressed on progenitors and mature macrophages. Tie-2 is an angiopoietin receptor tyrokine kinase implicated in endothelial cell functions, which can also be present on selected macrophages. The CSF-1R+ EM progenitors arise from a Tie2+ cellular pathway that eventually gives rise to the majority of resident macrophage populations in most adult tissues [40]. Foetal liver stromal macrophages facilitate erythropoiesis by poorly characterised trophic interactions [41]. Apart from capturing membrane-bound phosphatidyl serine (PS) + erythrocyte nuclei for digestion, these F4/80+ macrophages bind clusters of developing erythroblasts through a divalent cation-dependent, non-phagocytic receptor selectively expressed by stromal macrophages [42]. Adhesion is mediated by alpha v beta 1 integrin (very late antigen-4, VLA-4) on erythroblasts and vascular cell adhesion molecule-1 (VCAM-1) on central macrophages [43], before erythrocytes are released into the foetal circulation. Foetal liver macrophages lose their haemopoietic properties after birth and transition into non-stromal macrophages, resembling nascent Kupffer cells, the mature macrophages of the liver.

**Fig. 4.**
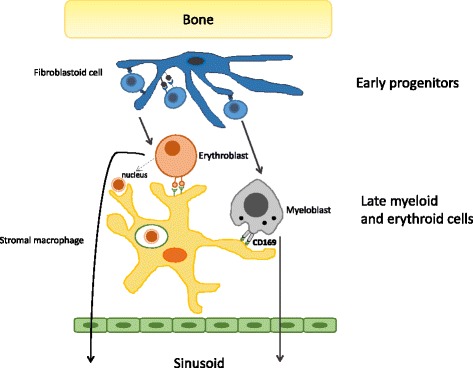
F4/80+ stromal macrophages in the bone marrow play a trophic role in haemopoiesis. Haemopoietic stem cells (HSC) associate with mesenchymal stromal cells in a specialised niche in the bone marrow during the early stages of haemopoiesis. After proliferation and differentiation, erythroblasts and myeloblasts associate with stromal F/80+ macrophages, forming haemopoietic islands with central macrophages. These stromal macrophages express non-phagocytic adhesion molecules, a divalent cation-dependent haemagglutinin and the sialic acid recognition receptor Siglec1 (CD169), which retain these committed haematopoietic cells for poorly defined trophic support, before they are ready for release into the circulation. In addition these stromal macrophages ingest erythroid nuclei and recycle Fe

In the adult bone marrow of mouse and human (Fig. 4), stromal macrophages at the centre of haematopoietic clusters continue to support the differentiation of erythrocytes and also myeloid leukocytes, including monocytes, by unknown surface and secreted mediators. Such islands were described by Bessis [44], and have been repeatedly observed by subsequent investigators, but have not received the attention they deserve. These mature phagocytic and trophic macrophages are relatively radio-resistant and are often overlooked as part of the haemopoietic stroma. Although it is not known how these macrophages signal to developing haemopoietic cells, they do specifically express adhesion molecules that mediate their interactions with them. In addition to the receptor described above for erythroblasts, they acquire CD169, a sialic acid-recognition molecule also known as sialoadhesin or SIGLEC-1. This non-phagocytic adhesion molecule of stromal macrophages is localised at attachment sites of developing neutrophils and eosinophils, but not erythroid cells [45]. CD169 regulation also plays a role in the release of haemopoietic cells into the circulation [8]. Haemopoietic stem cells associate with stromal mesenchymal cells, before passing to stromal macrophages, which also ingest and degrade erythroid nuclei, and store iron for re-use in erythropoiesis. Apart from the stromal macrophages associated with haemopoiesis, bone marrow contains monocyte progenitors, promonocytes, osteoclasts and unfused stellate macrophages on bone surfaces. Osteoclasts may arise directly from embryonic sources as well as from blood monocytes, as shown by parabiotic experiments.

## Spleen macrophages contribute to haemopoietic cell turnover and both innate and adaptive immunity

**Fig. 5.**
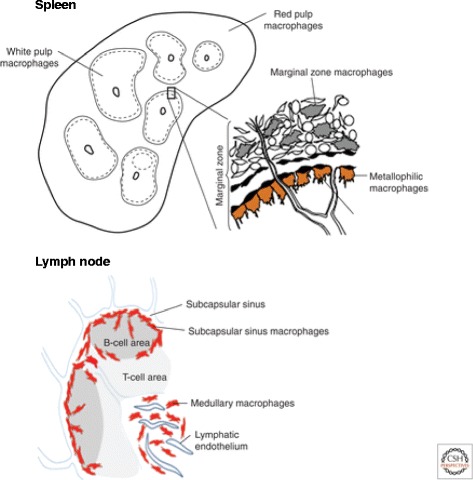
Macrophages in different regions of the mouse spleen and lymph node perform distinct functions in immunity and haemopoietic cell turnover. Schematic representation of regional differences of splenic macrophages in the red and white pulp, as well as the marginal zone. Marginal zone metallophils line vascular sinuses. Lymph nodes contain an analogous population that lines the subcapsular sinus. See text for further details. From [113], with permission

In the spleen, distinct macrophage subpopulations are present in discrete anatomical compartments, the red and white pulp regions, separated by a marginal zone (Fig. 5). This single organ combines functions of senescent erythroid and myeloid cell clearance, storage and production in the red pulp, with innate and acquired immunological responses to microbial and other antigens in the marginal zone and white pulp, illustrating the distinct adaptations of macrophages in each compartment. Red pulp macrophages clear effete blood cells by incompletely understood mechanisms, which may involve complement and PS recognition. They recycle iron [46] and catabolise haem [47], an inducer of Spi-C, a transcription factor found also in other macrophages implicated in erythrocyte turnover. In the mouse red pulp, there is also production of monocyte/macrophages which can be recruited to other peripheral organs [48].

The marginal zone of mouse spleen develops postnatally and contains a distinct metallophilic CD169+ subpopulation of macrophages responsible for sinusoidal immunity and interactions with DC [49] and the antibody-producing B lymphocytes that are an important component of the immune cell population of the spleen [50]. An outer, more phagocytic MARCO scavenger receptor + macrophage population is important in the >capture of polysaccharide-rich pathogens. Marginal zone macrophages are important in defence against bacterial infection in the circulation, and delayed maturation of these cells in newborn mice and human infants, or splenectomy in adults, results in vulnerability to infection. The white pulp macrophages and DC express CD68+, a pan-macrophage endosomal antigen which is strikingly upregulated by phagocytosis; these antigen processing and presenting cells migrate to splenic white pulp and to lymph nodes following antigen stimulation. The white pulp resembles other T-cell-rich lymphoid tissues such as Peyer’s patch, in that macrophages express little or no F4/80 antigen.

## Macrophages contribute to the induction of adaptive immunity in lymph nodes

The subcapsular sinus of lymph nodes (Fig. 5) receives afferent lymph and DC bearing antigens, for activation of B and T lymphocytes of the adaptive immune system. It is lined by sinusoidal CD169+ macrophages, analogous to the marginal metallophilic cells in spleen, which transfer captured antigens to DC in a cell relay to activate lymphocytes [51].

Lymph nodes are a graveyard for macrophages, which turn over locally, unlike DC, which enter efferent lymph and the systemic circulation. Medullary macrophages express F4/80 and CD68, strongly enhanced by phagocytosis of apoptotic lymphocytes. Complement receptors on a non-macrophage population of follicular cells with a distinctive dendritic morphology contribute to the interactions of B lymphocytes with antigen-presenting cells (APC) in germinal centres, the site of B lymphocyte proliferation and maturation in response to infection.

## Macrophages in the gastrointestinal tract interact with gut microbial flora

**Fig. 6.**
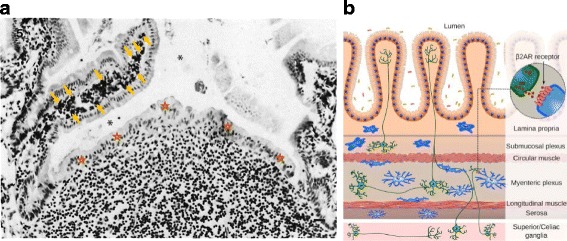
Gut macrophages populate the lamina propria and the myenteric plexus and interact with the microbiome and immune cells as well as the epithelium, smooth muscle and nerves. **a** Lamina propria macrophages in the mouse small intestine express abundant F4/80 antigen, indicated by *arrows*. The T-cell-rich Peyer’s patch and dome epithelium (*stars*) in the centre of the micrograph are devoid of F4/80 expression. Intestinal lumen, *asterisks*. From [114], ©Hume et al., 1983. Originally published in *The Journal of experimental medicine*. http://doi.org/10.1084/jem.158.5.1522. **b** Schematic representation of intestinal cross section to show interactions of macrophages (*blue*) with myenteric and autonomic nervous system projections (*green*). The inset shows the nerve ending releasing neurotransmitter which is recognized by β2 adrenergic receptors (β2AR) on the macrophage. From [54], reprinted from Cell, 164, Gabanyi I, Muller PA, Feighery L, Oliveira TY, Costa-Pinto FA, Mucida D, Neuro-immune Interactions Drive Tissue Programming in Intestinal Macrophages, 378,©2016, with permission from Elsevier

Resident macrophages are present throughout the gastrointestinal tract and play a complex role in the different specialised regions associated with digestion and absorption of nutrients, peristalsis, fluid balance and, above all, the symbiotic interactions with microbial flora, mucosal immunity and host defence against pathogens. We concentrate here on the small and large bowel, which contain the largest F4/80 + macrophage population in the body [52], mostly in the lamina propria (Fig. 6), as well as heterogeneous APC with poorly defined macrophage and DC characteristics. In the steady state, macrophages represent a mixture of embryo- and bone marrow-derived cells, responding to high local turnover of tissue-resident macrophages [28]. Macrophages and DC contribute to mucosal immunity in various ways. The F4/80 antigen has been implicated in oral tolerance to selected food antigens [53]; commensal bacteria in the lumen of the gut are, for the most part, shielded from direct contact with APC by mucus and an intact epithelium. APC, including macrophages, do extend cell processes into the gut lumen, to sample microbial flora and their products, which elicit immune responses in the case of infectious pathogens and are closely associated with high turnover of epithelium in crypts. Lamina propria macrophages migrate continually along the base of epithelial cells as these undergo a gradient of differentiation from stem cells towards the tip of intestinal villi [54]. The adaptation of macrophages and DC to the specialised microenvironment of the intestine is considered in the context of local imprinting by the microbiome, epithelial diversity and lymphocyte heterogeneity by Mucida and colleagues [55].

Macrophages in the smooth muscle layer interact with enteric neurons of the autonomic nervous system to enhance tissue protective responses to perturbation [54] and to enhance motility [56]. Macrophages expressing CX3CR1, a chemokine receptor which is characteristic of tissue-resident cells, are important in counteracting inflammatory responses in the gut by microbial products and cytokines such as IL-22 released by activation of specialized innate lymphoid cells (the so-called ILC2/3 lymphoid cells) [57]; the uptake of apoptotic cells also induces an anti-inflammatory phenotype through TGF beta and IL-10 production by macrophages, supplemented by cytokines produced by local fibroblasts.

Inflammatory bowel diseases affecting both the small and large intestine promote extensive recruitment of monocytes and activation of macrophages. Crohn’s disease is associated with genetic disorders of autophagy and with granuloma formation, including the appearance of multinucleated giant cells, products of monocyte-derived macrophage fusion. Ulcerative colitis involves loss of protective barrier to infection by commensals and pathogenic bacteria and is characterised by persistent influx of polymorphonuclear leukocytes (PMN) and macrophage-rich chronic inflammation, accompanied by tissue destruction and fibrosis. Other examples of important functions of intestinal macrophages include intestinal parasitic infection which promotes Th2-mediated alternative (M2) macrophage activation, parasite expulsion and fibrosis, as well as HIV-1-induced enteropathy, due to depletion of Th1 lymphocytes and deficient classic (M1) activation.

## Kupffer cells have immune, clearance and metabolic functions in the liver

**Fig. 7.**
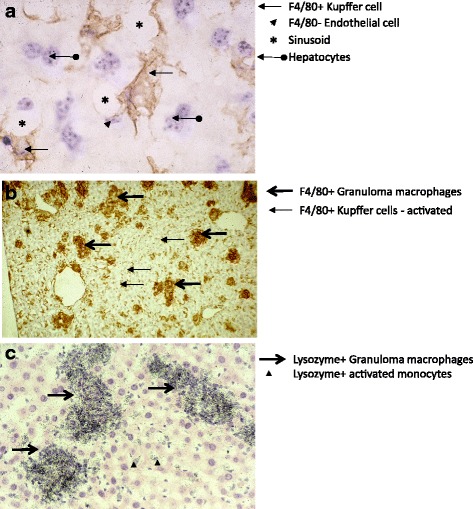
Kupffer cells, monocytes and macrophages interact with sinusoidal epithelium, hepatocytes and immune cells. **a** Normal mouse liver. Sinusoids (*asterisks*) are bordered by F4/80+ Kupffer cells (*arrows*) and F4/80 negative endothelial cells (*arrowheads*), in close proximity to hepatocytes, which are often binucleated (*broken arrow*). **b**, **c** Granuloma formation. Macrophages in granulomas induced by the mycobacterial vaccine Bacille Calmette Guérin (BCG) express F/80 antigen (*bold arrows*) on a background of activated Kupffer cells (*slender arrows*) and activated monocytes (**b**); BCG-induced recruitment of activated monocytes in sinusoids (*triangles*) and M1 activated macrophages in granulomas (*arrows*), which express lysozyme strongly and uniformly, detected by in situ hybridisation. See [115] for further details

Kupffer cells, the resident macrophages of the liver, are F4/80+ phagocytes (Fig. 7a) and express a distinct tissue-resident macrophage phenotype, downregulating CR3 and expressing CRIg, a tissue-specific complement receptor, as well as a liver-specific lectin for alpha-galactosyl ceramide [58], reflecting their function in innate recognition and adhesion. Kupffer cells express the receptors CD206 and SR-A, responsible for clearance of mannosylated glycoconjugates [59] and of selected polyanionic ligands such as calciprotein particles [60], respectively. Consistent with their common sinusoidal location, these major clearance functions of Kupffer cells are shared with hepatic sinusoidal endothelial cells, which are F4/80 negative, perhaps reflecting a common anatomic developmental origin.

Microbial products from the gut drain into the liver via mesenteric lymph nodes and the portal vein; repeated exposure to lipopolysaccharides (LPS) derived from bacterial walls of gut microbes desensitize and inactivate the Kupffer cells, so that host resistance to infection depends on newly recruited monocytes [61]. Indeed, Bleriot and colleagues have shown that infection by *Listeria monocytogenes* induces necroptosis of embryonic-derived Kupffer cells and their replacement by monocytes from bone marrow through sequential responses to macrophage loss [62].

Lipid and iron homeostasis represent other important metabolic aspects of macrophage functions in liver and their interactions with hepatocytes and the intestine. Ferroportin, important for iron export from Kupffer cells, hepatocytes and enterocytes, is inhibited by hepcidin [63]. Through their scavenger receptors for modified plasma lipoproteins, endocytic receptors for plasma transferrin and catabolism of senescent erythrocytes, Kupffer cells provide lipid ligands and iron for hepatocyte biosynthesis and secretion into blood. Intracellular stores can exceed Kupffer cell degradative capacity, resulting in lipid foam cell formation and ferritin accumulation.

Interactions of hepatocytes and macrophage-derived cytokines such as IL-6 are important in the early response to systemic inflammation, in which the so-called acute phase plasma proteins, including proteins of the complement cascade, are produced by the liver to combat the infection, as well as in metabolic responses to chronic inflammation and malignancy. Granuloma formation in the liver accompanies systemic chronic infections such as *Mycobacterium bovis* (BCG), an inducer of M1 macrophage-rich lesions (Fig. 7b, c), and schistosome egg deposition, which induces M2 macrophage-rich granulomas. Apart from characteristic phenotypic changes in these monocyte-derived structures, strongly F4/80+ granuloma macrophages upregulate the synthesis of lysozyme, a potent microbicidal enzyme which is poorly expressed in Kupffer cells and other resident tissue macrophages.

## Peritoneal macrophages may serve as the guardians of the abdominal serous cavity

Much of our knowledge of macrophage cell and molecular biology derives from ex vivo studies of murine macrophage peritoneal populations. These can be readily purified by adhesion and cultivated in vitro after washout of the peritoneal cavity; cells can be obtained in different functional states as unstimulated, resident cells, as “elicited” or “inflammatory exudate” cells after injection of sterile agents such as thioglycollate broth, polyacrylamide beads, zymosan particles, or bacterial LPS, or as immunologically activated M1 or M2 macrophages by specific antigen challenge, after infection. Peritoneal macrophages migrate rapidly to draining lymph nodes after intraperitoneal stimulation. Yet, in spite of numerous studies the functions of peritoneal macrophages remained unknown until recently. In remarkable studies, Kubes and colleagues demonstrated by intravital microscopy that F4/80 + resident peritoneal macrophages are recruited to the liver after sterile injury, for example by local laser-induced hepatic necrosis [64]. Earlier studies [65, 66] had demonstrated that a subpopulation of large resident peritoneal macrophages selectively express the transcription factor GATA-6; the Kubes group showed that these macrophages represent an independent reserve population of mature macrophages which can be rapidly mobilised, acquiring characteristics of M2 macrophages which promote repair after hepatic cell death. Thus, in pathology the liver can contain several macrophages of distinct origin, namely Kupffer cells of embryonic origin for homeostatic functions in the steady state, monocytes delivered from the bone marrow for host defence, and resident GATA-6+ peritoneal macrophages as a reservoir to restore tissue integrity after acute injury. This concept can be extended to other organs in the abdomen and to serosal populations in the pleural and pericardial cavities.

## Lung macrophages are the guardians of the airway

The lung contains alveolar macrophages of embryonic origin, which turn over independently of the bone marrow; alveolar macrophage production and maturation depend on the transcription factor PPAR gamma. In addition, the airway contains antigen-responsive bronchial DC and interstitial macrophages. Monocytes are recruited late in adult life to replenish alveolar macrophages and in response to inflammation. Alveolar macrophages play an essential part in clearance of particles, microbes, dust and pollutants and in the regulation of surfactant proteolipid turnover through local secretion of GM-CSF, in whose absence surfactant proteins accumulate in the alveoli and compromise lung function. Alveolar macrophages are rounded, loosely adherent cells and display a distinctive phenotype from other lung or tissue macrophages; they are F4/80 dim, CR3 low or absent, and express high levels of CD206, which recognizes microbial carbohydrates, and the scavenger receptors SR-A and MARCO for clearance of particles. The oxygen-rich environment may generate ligands for these scavenger receptors.

During allergic asthma, IL-4 and IL-13 production by antigen-activated Th2 lymphocytes induces M2 activated macrophages; these contribute to the further influx of monocytes by release of selected chemokines, generate arachidonate metabolites which promote bronchospasm by airway smooth muscle, goblet cell secretion and fibrosis [19, 67]. By contrast, monocyte-derived M1 macrophages induced by Interferon gamma in tuberculosis, for example, contribute to pro-inflammatory cytokine production, generation of nitric oxide- and oxygen-derived metabolites, and microbial killing; these products are responsible for host cell death, caseation, cavitation, haemoptysis and fibrosis, important complications to which macrophage secretory products such as collagenase and elastase contribute. Both M1 and M2 chronic inflammatory responses can result in macrophage fusion and giant cell formation. Granuloma formation depends on monocyte recruitment, cell activation, CR3 function and membrane-bound TNF.

## Macrophages play an important part in brain development as well as injury and neurodegeneration

**Fig. 8.**
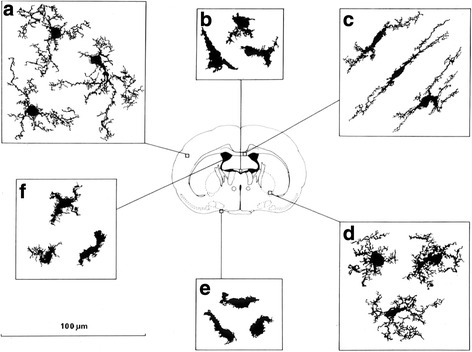
Morphological heterogeneity of F4/80+ microglia in the adult mouse brain. F4/80+ microglia are present in large numbers in all major divisions of the brain, but are not uniformly distributed. There is a more than five-fold variation in the density of immunostained microglial processes between different regions. More microglia are found in gray than in white matter. Microglia vary in morphology depending on their location. Compact cells are rounded, sometimes with one or two short thick limbs, bearing short processes. They resemble Kupffer cells of the liver and are found exclusively in sites lacking a blood–brain barrier. Longitudinally branched cells are found in fibre tracts and possess several long processes which are usually aligned parallel to the longitudinal axis of the nerve fibres. Radially branched cells are found throughout the neuropil. They can be extremely elaborate and there is wide variation in the length and complexity of branching of the processes. The systematic variation in microglial morphology provides evidence that these cells are exquisitely sensitive to their microenvironment. See [38] for further details. Camera lucida drawing courtesy of L.J. Lawson and V.H. Perry. The different panels show: **a** microglia in the cortex; **b** macrophages of the subfornical organ, one of the circumventricular organs lacking a blood brain barrier; **c** microglia of the white matter; **d** microglia in the ventral pallidum, one of the most densely populated regions of the central nervous system (note the smaller territories of the microglia); **e** macrophages of the meninges; **f** macrophages of the choroid plexus. In addition, the central nervous system contains perivascular macrophages which express F4/80 as well as the clearance receptors SR-A and CD206, which are downregulated in resident microglia in normal brain (not shown)

The brain contains several distinct resident populations of microglia and other macrophages, which have aroused considerable historical and current interest [68–70]. During development, before and after birth, cells of embryonic origin enter the central nervous system via the forming blood–brain barrier to remove apoptotic neurons, after differentiating into microglia, which are the main resident macrophages in the brain. These patrol the neuropil actively, regulate neurogenesis and sculpt synapses. This process occurs through the phagocytic receptor CR3 [71], which is highly expressed by microglia, as is F4/80. Recent studies by Squarzoni and colleagues have shown that microglia are able to modulate the outgrowth of dopaminergic neurons in the developing forebrain and the laminar positioning of subsets of neocortical interneurons [72]. Microglia become arborized in the neuropil environment (Fig. 8), turn over slowly in situ and remain as a morphologically heterogeneous network in grey and white matter throughout adult life. They react to injury and round up and aggregate during gliosis—a scarring response of glial cells—but their maintenance and functions in the adult steady state are not clear. Bruttger and colleagues have shown that after ablation, microglial repopulation is driven by local self-renewing progenitors in response to IL-1R signalling [73]. CCR2, the major chemokine receptor for the recruitment of monocytes of bone marrow origin, contributes to the pool of macrophages and microglia in the central nervous system after traumatic brain injury [74, 75] and in brain malignancy [76]. Resting microglia are characterised by extensive membrane processes which may perform additional housekeeping functions, for example in homeostasis of neurotransmitters such as glutamate, which they metabolise actively [77].

Astrocytes can also be induced to phagocytose dying cells, as well as interacting trophically with microglia. Following injury and a range of neurodegenerative diseases, a subset (A1) of neurotoxic reactive astrocytes is induced by activated microglia through secretion of IL-1alpha, TNF and C1q, a component of the classic complement cascade; A1 astrocytes lose their neuronal survival, outgrowth, synaptogenesis and phagocytic activity and induce the death of neurons and oligodendrocytes [78]. In mouse models of Alzheimer’s disease, complement and inappropriately activated microglia mediate synapse loss; complement component C1q is necessary for the toxic effects of soluble beta-amyloid oligomers on early synapse loss and hippocampal long-term potentiation (which is thought to reflect the processes underlying memory) [79]. Studies by Fonseca et al. [80] have shown that activated microglia, and not neurons or peripheral macrophages, are the source of C1q in the ageing and neurodegenerating brain of mice. The interrelation between microglia, complement and clearance of soluble beta amyloid is complicated by CR3-induced secretion of proteolytic activity, independent of phagocytosis, which regulates A beta levels [81]. A complement–microglial axis has also been reported to drive synapse loss in viral neuroinvasive disease [82]. Additionally, the macrophage/microglial molecule TREM2, which triggers intracellular tyrosine kinase phosphorylation (Fig. 2), senses anionic lipids known to associate with neuronal fibrillar A beta, sustaining the microglial response [83].

A distinct subpopulation of stellate perivascular macrophages in the brain expresses CD206 and SR-A clearance receptors, which are downregulated in resident microglia unless the microglia are activated by local inflammation or excitotoxin injury. These receptors may limit the diffusion of potential ligands into the neuroparenchyma if they cross the vascular bed. Some microglia, for example in the paraventricular regions, are outside the blood–brain barrier and express the sialic acid-recognition receptor CD169 [84], described above, which depends on a circulating plasma protein, possibly type 1 interferon, for its induction. This observation suggests that the blood–brain barrier plays a role in regulating microglial responses to proinflammatory cytokines in the systemic circulation. Finally, macrophages form a network in the leptomeninges [85], adjacent to a newly described lymphatic clearance system [86], and are prominent in the choroid plexus, where they are closely associated with epithelial cells responsible for secretion of cerebrospinal fluid.

In the peripheral nervous system, macrophages play a major role in myelin phagocytosis and proteolipid breakdown. Macrophage activation by injury and conditions such as T-cell-driven multiple sclerosis promote myelin catabolism through enhanced secretion of neutral proteinases such as plasminogen activator and elastase, to which myelin is exquisitely sensitive. Both resident and recruited cells contribute to degeneration and repair through their secretory and phagocytic activities [87, 88]. Alternatively activated (M2) macrophages and the IL-4 pathway through which they are activated have been utilised in the response to neuronal injury and the process of repair [89, 90]. Macrophages interact with both cholinergic [91] and adrenergic [92] pathways in the autonomic nervous system, for example in the gut, as noted above [93]

## Macrophages are a neglected homeostatic population in endocrine and reproductive organs

Macrophages are present in the anterior and posterior pituitary gland [94, 95], pancreas [96] and adrenal and thyroid glands [97]. In the posterior pituitary, electron microscopy revealed that the macrophages/microglia wrap around living neuronal processes and take up oxytocin/vasopressin- containing granules which accumulate in their phagolysosomes [95]. This suggests a role in hormone processing. Similar functions may be ascribed to macrophages in adrenal, thyroid [98] and pancreatic [99] endocrine homeostasis. Endocrine organs contain hormonal ligands for CD206; thyroglobulin naturally contains terminal residues for uptake and processing by its mannose recognition domains, whereas leutropin bears a sulphated ligand for the N-terminal cysteine-rich domain, which mediates clearance from the circulation by the liver [100]. Finally, monocyte and macrophage recruitment and pro-inflammatory and antimicrobial properties are selectively and potently downregulated by glucocorticosteroids, with the risk of enhancing susceptibility to infection.

Macrophages are prominent in the ovary during the oestrus cycle, especially in phagocytic clearance of dying cells in the corpus luteum, and in the testis, where non-macrophage Sertoli cells remove aberrant sperm. During mammary gland development macrophages play a role in controlling proliferation and branching of terminal epithelial buds, in part through CSF-1 and also through expression of chemokine receptors such as CCR2 and D6, which regulate their chemokine levels and recruitment [101]. Macrophages and the antibacterial enzyme lysozyme are prominent constituents of breast milk. Finally, they play a major role in involution of the mammary gland by phagocytosis of apoptotic tissue, and by secretion of potent extracellular neutral proteinases such as collagenase and elastase.

## Macrophages contribute to electrical activity in the heart, to repair of myocardial infarction and to atherosclerosis in the cardiovascular system

Macrophages are present interstitially in heart, large arteries and veins, and as periarteriolar cells in the peripheral vascular system. They have an intimate relation with endothelium during inflammation, repair, infection, atherosclerosis and malignancy [20]. Cardiac macrophages of embryonic origin are progressively replaced by bone marrow-derived monocytes with age [102, 103]. In a recent study, Ensan and colleagues have shown that arterial macrophages in mice derive from both CX3CR1+ precursors in the embryonic yolk sac and from bone marrow-derived monocytes after birth [104]. In the adult steady state and after sepsis, arterial macrophages are maintained by local proliferation rather than monocyte recruitment. Survival of resident arterial macrophages depends on the interactions of fractalkine, the CX3CL1 ligand expressed by a variety of cellular sources, with its receptor on resident tissue macrophages.

In a remarkable study, Hulsmans and colleagues used optogenetic methods to show that macrophages facilitate electrical conduction in the heart [105]. Resident macrophages are abundant in the mouse and human AV nodes, and macrophage connexin 43 modulates the electrical activity of cardiomyocytes. Macrophage ablation induced AV block. During inflammation and repair, for example following myocardial infarction, recruited monocytes play a role in vascular permeability, angiogenesis and scar formation. In atherogenesis, monocytes bind to endothelium and accumulate cholesterol-rich low density lipoproteins, giving rise to foam cells. Cell breakdown and lipid accumulation give rise to atheroma formation. Platelets, smooth muscle cells, macrophages and fibroblasts all contribute to plaque stability, thromboembolism and plaque rupture. Libby and colleagues have emphasised the inflammatory network that links the brain, autonomic nervous system, bone marrow and spleen with atherosclerotic plaque and infarction [106]. In a mouse model of chronic heart failure after ligation of the coronary artery, Nahrendorf and colleagues have shown that distinct populations of steady state cardiac, monocyte-derived and locally sourced macrophages, distinct from M2 polarization, contribute to expansion of myocardial macrophage populations in non- ischaemic regions. This is sourced by local proliferation, CCR2-dependent recruitment, as well as extramedullary haemopoiesis, and depends on activation of the sympathetic nervous system [107].

## There is more to learn about how and where macrophages diversify

Tissue macrophages display remarkable versatility in adapting to the needs of the body, counteracting and limiting changes in their local and systemic environment. They constitute a two-edged sword in host protection and injury, but it is not clear without further study whether their plasticity reflects population changes (recruitment, proliferation versus programmed death, necrosis or emigration) and/or altered gene expression at the level of individual cells. As terminally differentiated cells, mature tissue macrophages express a limited capacity for replication, but high RNA and protein synthesis, as well as marked posttranslational modification, even indications of “trained memory”, when innate immune stimuli such as BCG, a mycobacterial vaccine, or zymosan particles, acting via Dectin-1, the beta-glucan receptor, prime macrophages for enhanced responses to a subsequent unrelated challenge [108]. They respond to their cellular environment through a range of surface, vacuolar and cytosolic sensors, in turn providing their neighbours and distant targets with contact and diffusible signals to control metabolism. Their phagocytic capacity is variable, and may even be undetectable, but provides a well-developed machinery to internalise, degrade and store cargo such as poorly degraded foreign particles. An intriguing study by Hidalgo and colleagues assessed the impact of phagocytosis on the phenotype of macrophages isolated from different tissues, utilising different receptors, opsonins and transcription factors, to ingest host-derived cargo after parabiosis [109]. While macrophages from different origins continued to express a tissue-specific phenotype, phagocytosis imprinted a distinct anti-inflammatory profile of enhanced CD206 and decreased IL-beta expression. This study elegantly illustrates the interplay between phagocytic activity and local tissue-derived factors in establishing macrophage heterogeneity.

We now know that tissue macrophage populations have a mixed embryonic and postnatal bone marrow origin, but the mechanisms by which diversification occurs during differentiation and activation are not understood. Extrinsic stimuli such as the microbiome and pathogens can induce a spectrum of modular changes in gene expression, depending on time and place; these require an interplay between extrinsic and intrinsic mechanisms, including cytokine regulation, selective adhesion, receptor signalling and import of transcription factors to accessible euchromatin. We cannot readily distinguish resident-tissue macrophages and recruited monocyte-macrophages once they co-exist in a common environment. It will be a challenge to compare the numbers and contribution of tissue-resident macrophages and recruited monocytes in subcompartments within and between different organs, and in tissue-inflammatory infiltrates. Finally, do they communicate among themselves locally and systemically, to regulate their production, activities and lifespan?

Although we have learned a great deal from genetic and cell culture experiments, it is essential to develop further methods to screen for novel functions within the native tissue microenvironment. The ability to reconstruct matrix composition [110] and organ-specific environments in vitro, in combination with induced pluripotent precursor technology, should make it possible to discover and validate more functions of tissue macrophages in health and disease.

## Additional files

Additional file 1: Properties of activated monocytes and macrophages [26]. (DOC 27 kb)

Additional file 2: Phenotypic heterogeneity of macrophages in tissues [1, 22, 37–39, 65, 66, 74, 122–134]. (DOC 106 kb)

## Abbreviations

APC: Antigen-presenting cell
BCG: Bacille Calmette Guerin vaccine
CCR2: C-C chemokine receptor, type 2
CR3: Complement receptor type 3
CSF-1: Colony stimulating factor, type 1
DC: Dendritic cells
GM-CSF: Granulocyte macrophage colony stimulating factor
LPS: Lipopolysaccharide
MPS: Mononuclear phagocyte system
PMN: Polymorphonuclear leukocyte
SIGLEC1: Sialic acid binding Immunoglobulin like lectin 1
SR-A: Scavenger receptor, class A
TGM2: Transglutaminase 2
