# Supplementary material for: Tissue macrophages: heterogeneity and functions
Source: BMC Biol. 2017 Jun 29;15:53. doi: 10.1186/s12915-017-0392-4 (PMC5492929; doi:10.1186/s12915-017-0392-4)
Supplement: Supplementary file 1 — Properties of activated monocytes and macrophages [26]. (DOC 27 kb) [file 12915_2017_392_MOESM1_ESM.doc]

1. Turnover. Activated macrophages are shorter lived and respond more readily to CSF-1 and other growth factors than resident cells.
2. Migration. Activated cells produce and respond to different chemokines; resident cells express CX3CR1, ligand fractalkine, whereas blood monocyte recruitment is mediated by CCR2, ligand CCL1(MCP).
3. Cell–cell interactions. Macrophage stimulation induces trophic support of cellular growth during development and repair, including angiogenesis. Monocyte recruited tumour-associated macrophages (TAM) promote tumour growth and metastasis (MAM), whereas myeloid-derived suppressor cells (MDSC), immature monocytic and granulocytic cells, suppress tumor infiltrating lymphocyte activities.
4. Monocyte/macrophages recognise and ingest microbes and cellular targets by various opsonic (antibody FcR, complement receptors) and non-opsonic plasma membrane phagocytic receptors (Scavenger R, lectins). Apoptotic cells are cleared by multiple receptors for phosphatidyl serine (PS) and other ligands. The expression and regulation of phagocytic activities vary among resident and activated cells, depending on the receptor ligand.
5. Plasma membrane and vacuolar TLR and cytosolic receptors(NLR, RLR ) sense microbial products and nucleic acids in cytoplasm, stimulating inflammasome assembly, caspase cleavage and release of IL-1 beta.
6. Receptor-mediated endocytosis by inflammatory macrophages can result in increased uptake of folates and other micronutrients.
7. Enhanced signalling of NFkB and other pathways results in nuclear import of transcription factors and altered gene transcription and enhancer access to chromatin.
8. Interferon gamma (innate and lymphoid TH1 cytokine product) activates monocyte/macrophages and upregulates i-NOS and reactive oxygen radicals in response to microbial stimuli, killing microbial and cellular targets. Classic/M1 activation.
9. Interleukins-4 and -13 (TH2 cytokines) induce alternatively activated (M2) macrophages which express increased MHCII, arginase, CD206 (mannose receptors) and transglutaminase 2.
10. M1 and M2 modules of gene expression co-exist in vivo, as part of a spectrum of phenotypes, rather than polar opposites.
11. Activated macrophages express pro-inflammatory cytokines (IL-12 and -18, TNF alpha, IL-6) as well as anti-inflammatory products, IL-10, transforming growth factor-beta, and prostaglandin E2, to limit activation.
12. Activated monocytes can induce clotting following injury and infection, contributing to diffuse intravascular coagulation.
13. Activated macrophages secrete urokinase, elastase and collagenase, neutral proteinases contributing to fibrinolysis, repair and matrix remodelling.
14. Chronic TH1 and TH2 cellular immune responses induce macrophage-rich granulomas, e.g. to tuberculosis and schistosome eggs, respectively. These contain multinucleated macrophage giant cells, resulting from fusion of monocytes/macrophages.
15. Macrophage metabolism (e.g. glycolysis versus oxidative phosphorylation) changes in parallel with M1/M2 polarisation of macrophages.

For further details see .
