# Supplementary material for: Tissue macrophages: heterogeneity and functions
Source: BMC Biol. 2017 Jun 29;15:53. doi: 10.1186/s12915-017-0392-4 (PMC5492929; doi:10.1186/s12915-017-0392-4)
Supplement: Supplementary file 2 — Phenotypic heterogeneity of macrophages in tissues [1, 22, 37–39, 65, 66, 74, 122–134]. (DOC 106 kb) [file 12915_2017_392_MOESM2_ESM.doc]

Immunocytochemistry of F4/80 and other macrophage-expressed antigens, combined with morphology, reveal multiple distinct macrophage subpopulations within individual organs. This is particularly evident in the central nervous system, but is also apparent in spleen and gut (discussed below), and indeed in all solid organs. The F4/80 plasma membrane antigen, combined with other membrane antigens, has made it possible to map mouse tissue macrophages during development, from its first appearance in mid-gestation throughout life, in normal mice and in models of disease. In situ analysis established that F4/80+ macrophages were not only markedly heterogeneous within individual organs, but were also closely associated with endothelia and epithelia . However, particular F4/80 negative macrophage-like cells, e.g. metallophilic macrophages in spleen, expressed other antigen stained by second generation monoclonal antibodies (mAbs) such as sialoadhesin (CD169, Siglec1) , which also detected selected F4/80+macrophages. Conversely, many F4/80+ macrophages did not express CD169, an early indicator of phenotypic heterogeneity. CD68, a widely used pan-macrophage marker is an intracellular, endosomal antigen and therefore less informative in pointing to potential interactions with neighbouring cells; as for all individual reagents, there are situations where this antigen is present on non-macrophages. The development of mAbs to the type 3 complement receptor (CR3, CD11b/CD18, a beta 2 integrin) confirmed that CR3 expression was heterogeneous in situ. It also illustrated the point that cells lacking CR3 rapidly upregulated its expression in vitro, one of several clues that culture conditions resulted in artefacts compared with expression within the tissue environment.

The F4/80 antigen turned out to be an excellent marker for macrophages in yolk sac, foetal liver, spleen and other tissues, including the developing brain . The human homologue of F4/80, EMR1, is not detected by present commercial antibodies on macrophages, but only eosinophils, also detected by F4/80 in the mouse. However, EMR2, a related adhesion G protein-coupled receptor, which is present on several myeloid leukocytes, provides excellent surface staining of human tissue macrophages . CD206 and CD163, a haptoglobin–haemoglobin clearance receptor , are well expressed on many subpopulations of human tissue macrophages. There is a clear need for more reagents suitable for FACS and immunocytochemical analysis of human as well as mouse tissue macrophages.

A different, more recent approach has employed intravital microscopic imaging and lineage fate mapping analysis, using transgenesis and fluorescent knock-in of markers such as GFP. Studies on the fractalkine receptor , the CSF-1 receptor (CD 115) and crosses with LysM-cre mice have made it possible to target selected myeloid cells which differ in expression of the CSF1-R and LysM genes. The transcription factor Myb has been utilised to define the origin of embryonic versus marrow macrophages; diphtheria toxin/DTR and tamoxifen/oestrogen receptor have been used to achieve conditional depletion, as have clodronate liposomes.

The advent of microarray and proteomic analysis has contributed a vast amount of information on the phenotypes of selected macrophage populations (e.g. ). Isolation of peritoneal macrophages with minimal exposure to culture conditions to avoid artefacts has identified genes that are expressed on selected resident macrophages, e.g. CD64, an FcR, and Mer-TK, and identified distinct chromatin changes associated with transcriptional control , and in subpopulations of large and small peritoneal macrophages . Other investigators identified an important role for GATA 6 and retinoids in peritoneal macrophage gene expression . To analyse macrophage populations in organs such as spleen and brain, for example, the cell isolation procedure is critical and may introduce artefacts compared with in situ analysis. In situ hybridisation has been used to study macrophage development in the embryo , but only to a limited extent in the adult. A particular difficulty is that many tissue macrophages are stellate or arborized and embedded in tissue, making them difficult to isolate by controlled enzyme digestion for FACS or RNA analysis. FACS analysis is more sensitive to detect less abundant gene products such as surface antigens, so is useful in the characterisation of lymphocytes. However, as more sensitive analytical methods become available and RNA-seq and other single cell methods become less costly, this situation should improve. Procedures that depend on nucleic acid amplification require careful controls for artefacts and validation by in situ expression.
